# 1-Methyl-2-[(*E*)-2,4,5-trimeth­oxy­styr­yl]­pyridinium iodide^1^

**DOI:** 10.1107/S1600536810043254

**Published:** 2010-11-06

**Authors:** Charoensak Mueangkeaw, Suchada Chantrapromma, Pumsak Ruanwas, Hoong-Kun Fun

**Affiliations:** a Crystal Materials Research Unit, Department of Chemistry, Faculty of Science, Prince of Songkla University, Hat-Yai, Songkhla 90112, Thailand; b X-ray Crystallography Unit, School of Physics, Universiti Sains Malaysia, 11800 USM, Penang, Malaysia

## Abstract

In the title compound, C_17_H_20_NO_3_^+^·I^−^, the cation exists in the *E* configuration. The pyridinium and benzene rings are close to coplanar, with a dihedral angle of 7.43 (12)° between them. The three meth­oxy groups of 2,4,5-trimeth­oxy­phenyl are essentially coplanar with the benzene plane, with C—O—C—C torsion angles of 1.0 (3), −1.9 (3) and 3.6 (3)°. A weak intra­molecular C—H⋯O inter­action generates an *S*(6) ring motif. In the crystal, the cations are stacked in columns in an anti­parallel manner along the *a* axis through π–π inter­actions, with a centroid–centroid distance of 3.7714 (16) Å. The iodide anion is situated between the columns and linked to the cation by a weak C—H⋯I inter­action.

## Related literature

For bond-length data, see: Allen *et al.* (1987 ▶). For related literature on hydrogen-bond motifs, see: Bernstein *et al.* (1995 ▶). For background to nonlinear optical properties and applications of pyridinium and quinolinium derivatives, see: Chanawanno *et al.* (2010 ▶); Chantrapromma *et al.* (2010 ▶); Ruanwas *et al.* (2010 ▶); Williams (1984 ▶). For related structures, see: Chanawanno *et al.* (2008 ▶); Fun *et al.* (2009 ▶); Kaewmanee *et al.* (2010 ▶). For the stability of the temperature controller used in the data collection, see: Cosier & Glazer (1986 ▶).
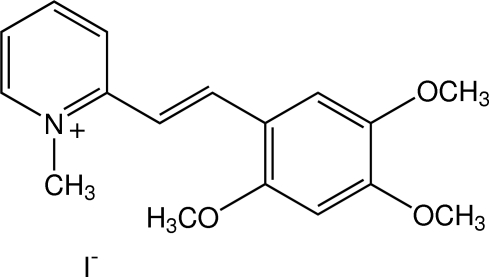


## Experimental

### 

#### Crystal data

C_17_H_20_NO_3_^+^·I^−^
*M* *_r_* = 413.24
Triclinic, 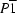

*a* = 8.9109 (1) Å
*b* = 10.3551 (1) Å
*c* = 10.8201 (2) Å
α = 113.382 (1)°
β = 109.243 (1)°
γ = 96.831 (1)°
*V* = 828.51 (2) Å^3^
*Z* = 2
Mo *K*α radiation
μ = 1.94 mm^−1^
*T* = 100 K
0.37 × 0.32 × 0.18 mm

#### Data collection

Bruker APEXII CCD area-detector diffractometer
Absorption correction: multi-scan (*SADABS*; Bruker, 2005 ▶) *T*_min_ = 0.534, *T*_max_ = 0.720
29408 measured reflections
4827 independent reflections
4686 reflections with *I* > 2σ(*I*)
*R*_int_ = 0.024

#### Refinement

*R*[*F* ^2^ > 2σ(*F* ^2^)] = 0.028
*wR*(*F* ^2^) = 0.084
*S* = 1.14
4827 reflections
203 parameters
H-atom parameters constrained
Δρ_max_ = 1.85 e Å^−3^
Δρ_min_ = −0.33 e Å^−3^

### 

Data collection: *APEX2* (Bruker, 2005 ▶); cell refinement: *SAINT* (Bruker, 2005 ▶); data reduction: *SAINT*; program(s) used to solve structure: *SHELXTL* (Sheldrick, 2008 ▶); program(s) used to refine structure: *SHELXTL*; molecular graphics: *SHELXTL*; software used to prepare material for publication: *SHELXTL* and *PLATON* (Spek, 2009 ▶).

## Supplementary Material

Crystal structure: contains datablocks global, I. DOI: 10.1107/S1600536810043254/is2612sup1.cif

Structure factors: contains datablocks I. DOI: 10.1107/S1600536810043254/is2612Isup2.hkl

Additional supplementary materials:  crystallographic information; 3D view; checkCIF report

## Figures and Tables

**Table 1:** Hydrogen-bond geometry (Å, °)

*D*—H⋯*A*	*D*—H	H⋯*A*	*D*⋯*A*	*D*—H⋯*A*
C6—H6*A*⋯O1	0.93	2.19	2.819 (3)	124
C14—H14*A*⋯I1^i^	0.96	3.03	3.992 (3)	177

Symmetry code: (i) 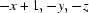.

**Fig. 1.**
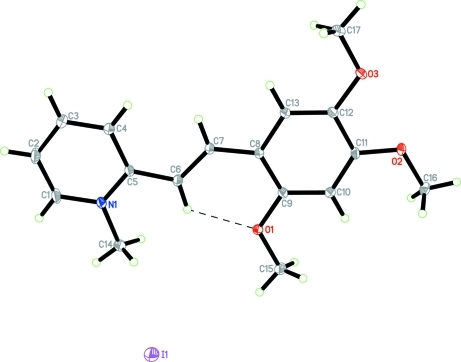
The molecular structure of the title compound, with 50% probability displacement ellipsoids and the atom-numbering scheme. Hydrogen bond was drawn as dashed line.

**Fig. 2.**
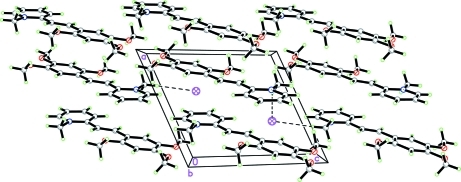
The crystal packing of the title compound viewed down the b-axis. Weak C—H···I interactions are shown as dashed lines.

## supplementary crystallographic information

### Comment

We have previously synthesized several pyridinium and quinolinium derivatives to study their antibacterial activities and non-linear optical (NLO) properties (Chanawanno *et al.*, 2008, 2010; Chantrapromma *et al.*, 2010; Fun *et al.*, 2009; Ruanwas *et al.*, 2010). During the course of antibacterial activities and NLO properties of synthetic compounds, the title pyridinium derivative was synthesized and its single crystal x-ray structural study was undertaken in order to establish the conformation of the various groups and the space group. The title compound (I) crystallized in the centrosymmetric triclinic *P*-1 space group and therefore (I) does not exhibit second order NLO properties (Williams, 1984). In addition (I) was also tested for antibacterial activities against the *Bacillus subtilis*, *Enterococcus faecalis*, *Staphylococcus aureus*, Methicillin-Resistant *Staphylococcus aureus*, Vancomycin-Resistant *Enterococcus faecalis*, *Pseudomonas aeruginosa*, *Salmonella typhi* and *Shigella sonnei*, and found inactive. Herein the crystal structure of (I) is reported.

Figure 1 shows the asymmetric unit of (I) which consists of a C_17_H_20_NO_3_^+^ cation and an I^-^ anion. The cation exists in the *E* configuration with respect to the C6═C7 double bond [1.350 (3) Å] with the torsion angle C5–C6–C7–C8 = -179.5 (2)°. The pyridinium and benzene rings are nearly coplanar with the ethenyl bridge with the dihedral angle between the pyridinium and benzene ring being 7.43 (12)°. The three methoxy groups of the 2,4,5-trimethoxyphenyl are essentially co-planar [C17–O3–C12–C13, C16–O2–C11–C10 and C15–O1–C9–C10 torsion angles of 1.0 (3), -1.9 (3) and 3.6 (3)°, respectively]. An intramolecular C6—H6A···O1 weak interactions generates an S(6) ring motif (Bernstein *et al.*, 1995) which helps to stabilize the planarity of the molecular structure. The methyl units of two methoxy groups at atoms C9 and C11 point towards whereas at atoms C11 and C12 point away from each other (Fig. 1). It is interesting to note that there is steric interaction between the methyl group of 1-methylpyridinium and the methoxy group at atom C9 but the intramolecular C6—H6A···O1 weak interaction which formed the S(6) ring motif is more preferable than the S(5) ring motif comparing to the structure which the 2,4,5-trimethoxyphenyl unit rotate 180° around the C7–C8 bond to form S(5) ring motif of C7—H7A···O1 weak interaction. The bond lengths of cation in (I) are in normal ranges (Allen *et al.*, 1987) and comparable to those in related structures (Chanawanno *et al.*, 2008; Fun *et al.*, 2009; Kaewmanee *et al.*, 2010).

In the crystal packing (Fig. 2), the cations are stacked in an anti-parallel manner along the *a* axis by π–π interactions with the *Cg*1···*Cg*2^ii^ distance of 3.7714 (16) Å [symmetry code: (ii) 1 - *x*, -*y*, 1 - *z*]; *Cg*1 and *Cg*2 are the centroids of N1/C1–C5 and C8–C13 rings, respectively. The iodide anions are located in the interstitials of the cations and linked to the cations by C—H···I weak interactions (Table 1).

### Experimental

The title compound (I) was prepared by mixing 1:1:1 molar ratio solutions of 1,2-dimethylpyridinium iodide (0.50 g, 2.13 mmol), 2,4,5-trimethoxybenzaldehyde (0.417 g, 2.13 mmol) and piperidine (0.21 ml, 2.13 mmol) in hot methanol (20 ml). The resulting solution was refluxed for 5 hr under a nitrogen atmosphere. The resultant solid which formed was filtered off and washed with diethyl ether. Orange block-shaped single crystals of (I) suitable for *x*-ray structure determination were recrystallized from methanol by slow evaporation at room temperature over a few weeks (m.p. 544-545 K).

### Refinement

All H atoms were positioned geometrically and allowed to ride on their parent atoms, with *d*(C—H) = 0.93 Å for aromatic and CH and 0.96 Å for CH_3_ atoms. The *U*_iso_ values were constrained to be 1.5*U*_eq_ of the carrier atom for methyl H atoms and 1.2*U*_eq_ for the remaining H atoms. A rotating group model was used for the methyl groups. The highest residual electron density peak is located at 1.46 Å from I1 and the deepest hole is located at 1.00 Å from C6.

### Crystal data

**Table:**

C_17_H_20_NO_3_^+^·I^−^	*Z* = 2
*M**_r_* = 413.24	*F*(000) = 412
Triclinic, *P*1	*D*_x_ = 1.656 Mg m^−^^3^
Hall symbol: -P 1	Melting point = 544–545 K
*a* = 8.9109 (1) Å	Mo *K*α radiation, λ = 0.71073 Å
*b* = 10.3551 (1) Å	Cell parameters from 4827 reflections
*c* = 10.8201 (2) Å	θ = 2.2–30.0°
α = 113.382 (1)°	µ = 1.94 mm^−^^1^
β = 109.243 (1)°	*T* = 100 K
γ = 96.831 (1)°	Block, orange
*V* = 828.51 (2) Å^3^	0.37 × 0.32 × 0.18 mm

### Data collection

**Table:**

Bruker APEXII CCD area-detector diffractometer	4827 independent reflections
Radiation source: sealed tube	4686 reflections with *I* > 2σ(*I*)
graphite	*R*_int_ = 0.024
φ and ω scans	θ_max_ = 30.0°, θ_min_ = 2.2°
Absorption correction: multi-scan (*SADABS*; Bruker, 2005)	*h* = −12→12
*T*_min_ = 0.534, *T*_max_ = 0.720	*k* = −14→13
29408 measured reflections	*l* = −15→15

### Refinement

**Table:**

Refinement on *F*^2^	Primary atom site location: structure-invariant direct methods
Least-squares matrix: full	Secondary atom site location: difference Fourier map
*R*[*F*^2^ > 2σ(*F*^2^)] = 0.028	Hydrogen site location: inferred from neighbouring sites
*wR*(*F*^2^) = 0.084	H-atom parameters constrained
*S* = 1.14	*w* = 1/[σ^2^(*F*_o_^2^) + (0.0418*P*)^2^ + 1.7349*P*] where *P* = (*F*_o_^2^ + 2*F*_c_^2^)/3
4827 reflections	(Δ/σ)_max_ = 0.001
203 parameters	Δρ_max_ = 1.85 e Å^−^^3^
0 restraints	Δρ_min_ = −0.33 e Å^−^^3^

### Special details

**Table:**

Experimental. The crystal was placed in the cold stream of an Oxford Cryosystems Cobra open-flow nitrogen cryostat (Cosier & Glazer, 1986) operating at 100.0 (1) K.
Geometry. All esds (except the esd in the dihedral angle between two l.s. planes) are estimated using the full covariance matrix. The cell esds are taken into account individually in the estimation of esds in distances, angles and torsion angles; correlations between esds in cell parameters are only used when they are defined by crystal symmetry. An approximate (isotropic) treatment of cell esds is used for estimating esds involving l.s. planes.
Refinement. Refinement of F^2^ against ALL reflections. The weighted R-factor wR and goodness of fit S are based on F^2^, conventional R-factors R are based on F, with F set to zero for negative F^2^. The threshold expression of F^2^ > 2sigma(F^2^) is used only for calculating R-factors(gt) etc. and is not relevant to the choice of reflections for refinement. R-factors based on F^2^ are statistically about twice as large as those based on F, and R- factors based on ALL data will be even larger.

### Fractional atomic coordinates and isotropic or equivalent isotropic displacement parameters (Å^2^)

**Table:**

	*x*	*y*	*z*	*U*_iso_*/*U*_eq_	
I1	0.63632 (2)	0.272468 (17)	0.263674 (17)	0.01981 (6)	
O1	0.1870 (2)	0.19738 (18)	0.43228 (18)	0.0142 (3)	
O2	0.0422 (2)	0.40374 (18)	0.85063 (19)	0.0141 (3)	
O3	0.1169 (2)	0.20276 (19)	0.92393 (19)	0.0152 (3)	
N1	0.3476 (2)	−0.2139 (2)	0.1812 (2)	0.0116 (3)	
C1	0.3923 (3)	−0.3317 (3)	0.1060 (3)	0.0164 (4)	
H1A	0.3941	−0.3480	0.0156	0.020*	
C2	0.4348 (3)	−0.4269 (3)	0.1614 (3)	0.0179 (4)	
H2A	0.4628	−0.5085	0.1084	0.022*	
C3	0.4351 (3)	−0.3990 (3)	0.2983 (3)	0.0157 (4)	
H3A	0.4656	−0.4611	0.3387	0.019*	
C4	0.3902 (3)	−0.2792 (2)	0.3738 (2)	0.0133 (4)	
H4A	0.3916	−0.2604	0.4657	0.016*	
C5	0.3422 (3)	−0.1847 (2)	0.3140 (2)	0.0105 (3)	
C6	0.2895 (3)	−0.0589 (2)	0.3851 (2)	0.0118 (4)	
H6A	0.2727	0.0048	0.3442	0.014*	
C7	0.2639 (3)	−0.0305 (2)	0.5083 (2)	0.0109 (3)	
H7A	0.2832	−0.0967	0.5456	0.013*	
C8	0.2103 (3)	0.0895 (2)	0.5905 (2)	0.0101 (3)	
C9	0.1697 (3)	0.2006 (2)	0.5538 (2)	0.0109 (4)	
C10	0.1146 (3)	0.3083 (2)	0.6399 (2)	0.0117 (4)	
H10A	0.0887	0.3814	0.6148	0.014*	
C11	0.0984 (3)	0.3064 (2)	0.7627 (2)	0.0111 (4)	
C12	0.1396 (3)	0.1966 (2)	0.8021 (2)	0.0116 (4)	
C13	0.1940 (3)	0.0915 (2)	0.7170 (2)	0.0112 (4)	
H13A	0.2210	0.0193	0.7435	0.013*	
C14	0.3022 (3)	−0.1177 (3)	0.1125 (3)	0.0177 (4)	
H14A	0.3121	−0.1545	0.0198	0.027*	
H14B	0.3754	−0.0198	0.1771	0.027*	
H14C	0.1899	−0.1165	0.0960	0.027*	
C15	0.1497 (3)	0.3104 (3)	0.3933 (3)	0.0196 (5)	
H15A	0.1785	0.3010	0.3127	0.029*	
H15B	0.2124	0.4049	0.4769	0.029*	
H15C	0.0333	0.3012	0.3641	0.029*	
C16	0.0013 (3)	0.5189 (3)	0.8167 (3)	0.0163 (4)	
H16A	−0.0356	0.5804	0.8869	0.024*	
H16B	−0.0855	0.4769	0.7189	0.024*	
H16C	0.0977	0.5766	0.8214	0.024*	
C17	0.1636 (3)	0.0946 (3)	0.9660 (3)	0.0197 (4)	
H17A	0.1415	0.1061	1.0503	0.030*	
H17B	0.2798	0.1062	0.9907	0.030*	
H17C	0.1008	−0.0016	0.8853	0.030*	

### Atomic displacement parameters (Å^2^)

**Table:**

	*U*^11^	*U*^22^	*U*^33^	*U*^12^	*U*^13^	*U*^23^
I1	0.02761 (10)	0.01804 (9)	0.01905 (9)	0.00876 (6)	0.01258 (7)	0.01051 (7)
O1	0.0254 (8)	0.0137 (7)	0.0124 (7)	0.0114 (6)	0.0120 (6)	0.0092 (6)
O2	0.0208 (8)	0.0128 (7)	0.0159 (7)	0.0108 (6)	0.0126 (6)	0.0075 (6)
O3	0.0240 (8)	0.0171 (8)	0.0139 (7)	0.0115 (6)	0.0132 (6)	0.0102 (6)
N1	0.0147 (8)	0.0123 (8)	0.0100 (8)	0.0061 (6)	0.0071 (6)	0.0052 (7)
C1	0.0202 (10)	0.0179 (10)	0.0138 (9)	0.0106 (8)	0.0107 (8)	0.0054 (8)
C2	0.0220 (11)	0.0166 (10)	0.0176 (10)	0.0115 (8)	0.0115 (9)	0.0058 (8)
C3	0.0185 (10)	0.0143 (10)	0.0189 (10)	0.0093 (8)	0.0101 (8)	0.0089 (8)
C4	0.0171 (9)	0.0136 (9)	0.0131 (9)	0.0076 (7)	0.0082 (8)	0.0074 (8)
C5	0.0117 (8)	0.0107 (8)	0.0097 (8)	0.0043 (7)	0.0056 (7)	0.0041 (7)
C6	0.0151 (9)	0.0109 (9)	0.0110 (9)	0.0062 (7)	0.0066 (7)	0.0049 (7)
C7	0.0132 (8)	0.0101 (8)	0.0108 (9)	0.0052 (7)	0.0060 (7)	0.0046 (7)
C8	0.0122 (8)	0.0101 (8)	0.0098 (8)	0.0048 (7)	0.0061 (7)	0.0046 (7)
C9	0.0138 (9)	0.0114 (9)	0.0093 (8)	0.0050 (7)	0.0058 (7)	0.0052 (7)
C10	0.0149 (9)	0.0111 (9)	0.0120 (9)	0.0061 (7)	0.0070 (7)	0.0064 (7)
C11	0.0122 (8)	0.0107 (9)	0.0117 (9)	0.0053 (7)	0.0071 (7)	0.0041 (7)
C12	0.0138 (9)	0.0134 (9)	0.0109 (9)	0.0060 (7)	0.0072 (7)	0.0066 (7)
C13	0.0142 (9)	0.0115 (9)	0.0109 (9)	0.0055 (7)	0.0066 (7)	0.0062 (7)
C14	0.0277 (11)	0.0193 (10)	0.0157 (10)	0.0122 (9)	0.0133 (9)	0.0120 (9)
C15	0.0355 (13)	0.0173 (10)	0.0165 (10)	0.0149 (9)	0.0146 (9)	0.0125 (9)
C16	0.0205 (10)	0.0128 (9)	0.0216 (11)	0.0100 (8)	0.0128 (9)	0.0089 (8)
C17	0.0298 (12)	0.0228 (11)	0.0190 (11)	0.0142 (10)	0.0154 (9)	0.0152 (9)

### Geometric parameters (Å, °)

**Table:**

O1—C9	1.362 (2)	C7—H7A	0.9300
O1—C15	1.432 (3)	C8—C9	1.407 (3)
O2—C11	1.358 (2)	C8—C13	1.416 (3)
O2—C16	1.433 (3)	C9—C10	1.399 (3)
O3—C12	1.377 (3)	C10—C11	1.391 (3)
O3—C17	1.423 (3)	C10—H10A	0.9300
N1—C1	1.360 (3)	C11—C12	1.411 (3)
N1—C5	1.366 (3)	C12—C13	1.375 (3)
N1—C14	1.481 (3)	C13—H13A	0.9300
C1—C2	1.372 (3)	C14—H14A	0.9600
C1—H1A	0.9300	C14—H14B	0.9600
C2—C3	1.391 (3)	C14—H14C	0.9600
C2—H2A	0.9300	C15—H15A	0.9600
C3—C4	1.378 (3)	C15—H15B	0.9600
C3—H3A	0.9300	C15—H15C	0.9600
C4—C5	1.406 (3)	C16—H16A	0.9600
C4—H4A	0.9300	C16—H16B	0.9600
C5—C6	1.448 (3)	C16—H16C	0.9600
C6—C7	1.350 (3)	C17—H17A	0.9600
C6—H6A	0.9300	C17—H17B	0.9600
C7—C8	1.451 (3)	C17—H17C	0.9600
			
C9—O1—C15	118.29 (17)	C9—C10—H10A	119.8
C11—O2—C16	117.54 (18)	O2—C11—C10	124.67 (19)
C12—O3—C17	115.34 (17)	O2—C11—C12	115.43 (18)
C1—N1—C5	122.11 (19)	C10—C11—C12	119.89 (19)
C1—N1—C14	117.39 (19)	C13—C12—O3	125.15 (19)
C5—N1—C14	120.50 (18)	C13—C12—C11	119.29 (19)
N1—C1—C2	121.0 (2)	O3—C12—C11	115.53 (18)
N1—C1—H1A	119.5	C12—C13—C8	122.16 (19)
C2—C1—H1A	119.5	C12—C13—H13A	118.9
C1—C2—C3	118.8 (2)	C8—C13—H13A	118.9
C1—C2—H2A	120.6	N1—C14—H14A	109.5
C3—C2—H2A	120.6	N1—C14—H14B	109.5
C4—C3—C2	119.8 (2)	H14A—C14—H14B	109.5
C4—C3—H3A	120.1	N1—C14—H14C	109.5
C2—C3—H3A	120.1	H14A—C14—H14C	109.5
C3—C4—C5	121.0 (2)	H14B—C14—H14C	109.5
C3—C4—H4A	119.5	O1—C15—H15A	109.5
C5—C4—H4A	119.5	O1—C15—H15B	109.5
N1—C5—C4	117.30 (19)	H15A—C15—H15B	109.5
N1—C5—C6	118.57 (19)	O1—C15—H15C	109.5
C4—C5—C6	124.13 (19)	H15A—C15—H15C	109.5
C7—C6—C5	122.6 (2)	H15B—C15—H15C	109.5
C7—C6—H6A	118.7	O2—C16—H16A	109.5
C5—C6—H6A	118.7	O2—C16—H16B	109.5
C6—C7—C8	128.7 (2)	H16A—C16—H16B	109.5
C6—C7—H7A	115.6	O2—C16—H16C	109.5
C8—C7—H7A	115.6	H16A—C16—H16C	109.5
C9—C8—C13	117.62 (18)	H16B—C16—H16C	109.5
C9—C8—C7	126.06 (19)	O3—C17—H17A	109.5
C13—C8—C7	116.29 (18)	O3—C17—H17B	109.5
O1—C9—C10	122.56 (19)	H17A—C17—H17B	109.5
O1—C9—C8	116.78 (18)	O3—C17—H17C	109.5
C10—C9—C8	120.65 (19)	H17A—C17—H17C	109.5
C11—C10—C9	120.37 (19)	H17B—C17—H17C	109.5
C11—C10—H10A	119.8		
			
C5—N1—C1—C2	−0.2 (3)	C7—C8—C9—O1	−2.0 (3)
C14—N1—C1—C2	−179.5 (2)	C13—C8—C9—C10	−0.3 (3)
N1—C1—C2—C3	−1.5 (4)	C7—C8—C9—C10	178.1 (2)
C1—C2—C3—C4	1.3 (4)	O1—C9—C10—C11	179.8 (2)
C2—C3—C4—C5	0.6 (4)	C8—C9—C10—C11	−0.3 (3)
C1—N1—C5—C4	2.0 (3)	C16—O2—C11—C10	−1.9 (3)
C14—N1—C5—C4	−178.8 (2)	C16—O2—C11—C12	178.99 (19)
C1—N1—C5—C6	−178.5 (2)	C9—C10—C11—O2	−178.3 (2)
C14—N1—C5—C6	0.6 (3)	C9—C10—C11—C12	0.8 (3)
C3—C4—C5—N1	−2.2 (3)	C17—O3—C12—C13	3.6 (3)
C3—C4—C5—C6	178.4 (2)	C17—O3—C12—C11	−178.1 (2)
N1—C5—C6—C7	173.0 (2)	O2—C11—C12—C13	178.53 (19)
C4—C5—C6—C7	−7.7 (3)	C10—C11—C12—C13	−0.6 (3)
C5—C6—C7—C8	−179.5 (2)	O2—C11—C12—O3	0.1 (3)
C6—C7—C8—C9	1.7 (4)	C10—C11—C12—O3	−179.03 (19)
C6—C7—C8—C13	−179.9 (2)	O3—C12—C13—C8	178.2 (2)
C15—O1—C9—C10	1.0 (3)	C11—C12—C13—C8	0.0 (3)
C15—O1—C9—C8	−178.9 (2)	C9—C8—C13—C12	0.4 (3)
C13—C8—C9—O1	179.63 (19)	C7—C8—C13—C12	−178.1 (2)

### Hydrogen-bond geometry (Å, °)

**Table:**

*D*—H···*A*	*D*—H	H···*A*	*D*···*A*	*D*—H···*A*
C6—H6A···O1	0.93	2.19	2.819 (3)	124
C14—H14A···I1^i^	0.96	3.03	3.992 (3)	177

Symmetry codes:  (i) −*x*+1, −*y*, −*z*.
